# Adaptation needs of an intervention to facilitate tobacco cessation among men attending emergency departments in Nairobi, Kenya

**DOI:** 10.64898/2026.09.02.26361107

**Published:** 2026-09-06

**Authors:** Mari Armstrong-Hough, Thomas Kedera, Miriam Bosire, Melvine Obuya, Grace Soma, Jessica Rayo, Lorien Abroms, Christine Ngaruiya

## Abstract

**Background:**

Three-quarters of cancer deaths globally are in low- or middle-income countries. Cost-effective tobacco cessation interventions are critical to reducing cancer burden in these settings. Interventions that reach men, who are more likely to smoke and less likely to be engaged in primary care, are particularly important in Kenya, where 84% of smokers are men. Engaging men using a text-based mHealth program is a promising strategy to promote cessation. Text-based quit counseling using Text2Quit, a US-based mHealth intervention, has been demonstrated to be effective in high-income settings. We aimed to identify barriers to using Text2Quit among Kenyan men who smoke and optimize Text2Quit for Kenya.

**Methods:**

We invited men who smoke in Nairobi, Kenya to participate in intercept interviews using a Text2Quit prototype adapted for Kenya. A multinational team of tobacco experts, designers, Kenyan physicians, and implementation researchers carried out a rapid analysis of interview transcripts to identify barriers to its usability, relatability, and appeal. These barriers and proposed solutions were enumerated and ranked using nominal group technique with rank-order voting.

**Results:**

Thirteen men participated in interviews using a Text2Quit-Kenya prototype. Participants identified rigid response formats as a barrier to its usability, long text messages as a barrier to relatability, and confusion about cost as a barrier to its appeal. Twenty-six proposed solutions were identified by the nominal group technique process and five were prioritized based on importance to stakeholders and feasibility: 1) enable minor deviations in patient responses, 2) reduce length of messages, 3) clearly communicate that Text2Quit is free of charge, 4) provide brief in-person counseling at enrollment, including training on how to find and reply to texts and setting a quit date before texts begin, and 5) provide individualized follow up with users.

**Conclusions:**

Barriers to uptake of Text2Quit among Kenyan men who smoke are addressable through a systematic adaptation process.

## Full Text Availability

The license terms selected by the author(s) for this preprint version do not permit archiving in PMC. The full text is available from the preprint server.

